# The Association of Healthy Lifestyle Behaviors with Overweight and Obesity among Older Adults from 21 Countries

**DOI:** 10.3390/nu13020315

**Published:** 2021-01-22

**Authors:** Priscila Marconcin, Andreas Ihle, André O. Werneck, Elvio R. Gouveia, Gerson Ferrari, Miguel Peralta, Adilson Marques

**Affiliations:** 1 Faculty of Human Kinetics, University of Lisbon, 1495-751 Lisbon, Portugal; 2 Center for the Interdisciplinary Study of Gerontology and Vulnerability, University of Geneva, 1205 Geneva, Switzerland; andreas.ihle@unige.ch; 3 Swiss National Centre of Competence in Research LIVES—Overcoming Vulnerability: Life Course Perspectives, Lausanne and Geneva, 1022 Chavannes-près-Renens, Switzerland; 4 Department of Psychology, University of Geneva, 1205 Geneva, Switzerland; 5 School of Public Health, Universidade de São Paulo, São Paulo 01246-904, Brazil; andreowerneck@gmail.com; 6 Departamento de Educação Física e Desporto, Universidade da Madeira, 9020-105 Funchal, Portugal; erubiog@staff.uma.pt; 7 Interactive Technologies Institute, LARSyS, 9020-105 Funchal, Portugal; 8 Laboratorio de Ciencias de la Actividad Física, el Deporte y la Salud, Facultad de Ciencias Médicas, Universidad de Santiago de Chile, USACH, 71783-5 Santiago, Chile; gerson.demoraes@usach.cl; 9 CIPER, Faculty of Human Kinetics, University of Lisbon, 1495-751 Lisbon, Portugal; mperalta@fmh.ulisboa.pt (M.P.); amarques@fmh.ulisboa.pt (A.M.); 10 ISAMB, University of Lisbon, 1649-028 Lisbon, Portugal

**Keywords:** weight status, older adults, vulnerability, healthy lifestyle, physical activity, public policies

## Abstract

This study aimed to analyze the association of healthy lifestyle behaviors with overweight and obesity among Europeans aged 65+ years. Data were from the 2014 European Social Survey, analyzing 21 countries. Five lifestyle behaviors (physical activity, fruit and vegetable consumption, sleep quality, drinking alcohol, and smoking) were analyzed. Binary logistic regressions were performed. A total of 8938 participants (4099 men) 65 years and older, mean age—73.6 (SD: 6.6) presented prevalence of overweight of 42.3% (95% CI: 41.3 to 43.3) and obesity of 20.9% (95% CI: 20.1 to 21.8). Adopting five healthy behaviors was associated with lower odds of obesity (OR = 0.50, 95% CI: 0.39 to 0.63), but not overweight (OR: 0.93; 95% CI: 0.79 to 1.10). Physical activity (≥5 days/week) was the most protective behavior, reducing by 42% the odds of obesity. Sex moderated the association of fruits and vegetables consumption, alcohol use, and smoking with obesity. Strategies aiming to reduce obesity levels in older adults should focus on the promotion of multiple lifestyle health behaviors, particularly physical activity in order to decrease vulnerability risk in old age.

## 1. Introduction

Globally, in 2017, high body-mass index (BMI) caused 2.4 million (95% UI 1.6 million, 3.4 million) deaths and 70.7 million (95% UI 49.1 million, 94.9 million) disability-adjusted life years (DALYs) lost in females, and 2.3 million (95% UI 1.4 million, 3.4 million) deaths and 77.0 million (95% UI 49.7 million, 108.2 million) DALYs lost in males [1]. The prevalence of overweight (body mass index: BMI ≥ 25 kg/m^2^) and obesity (BMI ≥ 30 kg/m^2^) in European adults (≥50 years) is estimated to be about 60% and 19%, respectively [2]. Among older adults, these prevalences are known to be higher than among middle-aged and younger adults [2]. Obesity in the aging population is a factor of vulnerability since it leads to several impairments, such as physical and functional limitations [3], increasing rates of metabolic syndrome [4], and increased risk of mortality [5], while overweight does not seem to be associated with morbidity and mortality [6,7,8,9].

Increased overweight and obesity are accompanied by societal, demographic, and cultural changes, involving mainly the adoption of unhealthy lifestyle behaviors, such as insufficient physical activity practice, unfavorable dietary behavior, sleeping patterns, and consumption of some substances such as alcohol and tobacco [10,11]. On the other hand, an increase in fruit and vegetal consumption contributes to reducing adiposity among overweight and obese adults [12].

Recently, a study found that the prevalence of Europeans that achieved recommended levels of physical activity, diet, low alcohol consumption, not smoking, and good sleep quality is very low (only 5.8%) [13]. Although prior studies have explored the association of individual behavioral risk factors with overweight and obesity for adults [10,14], the association of healthy lifestyle behaviors with overweight and obesity in older adults warrants further investigation [14]. Even though not properly tested, previous studies were inconclusive in pointing whether the association of health behaviors with overweight and obesity changes according to sex [10]. Therefore, by addressing these gaps to contribute to the existing literature and advance this important field of research, the present study aimed to examine in more detail the association of healthy lifestyle behaviors with weight status among European older adults (aged ≥65 years), with a focus on potential sex differences.

## 2. Materials and Methods

### 2.1. Study Design and Participants

This cross-sectional study is based on the European Social Survey (ESS) 2014 database. The ESS is a multi-country survey administered in more than 30 countries to date. It is designed as a biennial survey, which aims to provide a unique and innovative resource on the (changing) social, political, and economic conditions in Europe. Participants were sampled by postal code, address files, social security registry data, population registers and/or telephone books. The questionnaire was translated by language experts, into the language spoken in each of the participating countries. The information was collected using a questionnaire, filled in through an hour-long, face-to-face interview. Informed consent was obtained before participants filled out the questionnaires. The study protocol subscribes to the Declaration on Professional Ethics of the International Statistical Institute. The study is in accordance with the Declaration of Helsinki. More information on the data collection can be found on the ESS website http://www.europeansocialsurvey.org/.

For the present study, only participants aged 65 years and older were included. Respondents without information in (1) more than two sociodemographic variables, (2) BMI, and (3) information regarding physical activity, eating habits, sleep quality, alcohol consumption, and smoking were also excluded. The final sample comprised 8938 participants ≥65 years (4839 women), from 21 countries (Austria, Belgium, Switzerland, Czech Republic, Germany, Denmark, Estonia, Spain, Finland, France, United Kingdom, Hungary, Ireland, Israel, Lithuania, Netherlands, Norway, Poland, Portugal, Sweden, and Slovenia).

### 2.2. Measures and Instruments

Social demographic variables such as sex, age, and years of fulltime education (recorded as low, middle, and high, according to the International Standard Classification of Education) [15] were collected. Marital status was defined by the question of whether they live with or without a husband/wife/partner. To determine the living place, those who indicated that they lived in a big city, the suburbs, or the outskirts of a big city were grouped into a new category named “urban areas”; those who responded that they lived in the countryside, in a village, or a house in the countryside were grouped into a category called “rural areas”.

Weight and height were self-reported. BMI was calculated using the standard formula (ratio of height and weight, expressed as kg/m^2^). The resulting score was classified following the National Health Service (NHS) guidelines [16] to determine whether the respondent was in a normal weight range (18.5 to 24.9 kg/m^2^), overweight range (25 to 29.9 kg/m^2^), and obese (≥30 kg/m^2^). Individuals with BMI below 18.5 kg/m^2^ were not included in our sample.

To assess physical activity, a single item was used, “On how many of the last 7 days did you walk quickly, do sports, or other physical activity for 30 min or longer?” The answers were classified according to the European Physical Activity guidelines [16,17] in (1) having attained the recommendation (30 min of at least moderate physical activity on 5 occasions per week) and (2) not having attained the physical activity recommended levels (<30 min of at least moderate physical activity on 5 occasions per week). This single question is an acceptable way to assess physical activity [18], and was previously used with the European Social Survey data analyses [19].

The consumption of fruit and vegetables was assessed by combining answers to two questions, “How often do you eat fruit, excluding drinking juice?” and “How often do you eat vegetables or salad, excluding potatoes?” As previously used with the European Social Survey data [13], the consumption of at least four portions of fruit or/and vegetables a day was considered as the cutoff point.

Sleep quality was recorded by the question “For how long, during the past week, was your sleep restless?” Response options were—“none or almost none of the time”, “some of the time”, “most of the time”, and “all or almost all of the time”. If participants answered that sleep was restless none or almost none of the time, it was considered as a healthy sleep behavior [13].

Alcohol consumption was assessed by asking participants to indicate from all beverages they consumed, the last time they drank alcohol on a weekday and the last time they drank alcohol on a weekend day. The guidelines used were from the United Kingdom Department of Health and not drinking excessively was defined as less than 14 units/week (112 g) for women and 21 units/week for men (168 g) [20].

Smoking behavior was self-reported. Response options were from “I have never smoked” to “I smoke every day”. Because there is no threshold of safety for smoking, responses were recoded into a current smoker (regularly or sometimes), and not smoking [13].

Based on physical activity, fruit and vegetable consumption, sleep quality, alcohol consumption, and smoking behavior, a healthy lifestyle score was created [13] and the participants were classified as presenting the five healthy behaviors or not.

### 2.3. Statistical Analysis

Descriptive statistics as a frequency or mean and confidence interval were calculated. The prevalence of each healthy lifestyle behavior, stratified by BMI for the total sample and by sex was estimated. Multivariable logistic regression was performed to analyse the association of different health behaviors and the cluster of healthy behaviors with overweight and obesity. Sex-interaction terms were inserted to verify the role of sex in the association between different behaviors and weight status. Stratified analysis by sex with adjusted models for age, civil status, education level, and living place were also conducted, considering the significant sex interactions. We adjusted the analysis for the whole sample for sex, age, civil status, education level, and living place. We used the Hosmer–Lemeshow test for goodness-of-fit in each model. Statistical analysis was performed using SPSS version 26. The significance level was set at *p* < 0.05.

## 3. Results

Overall, the prevalence of overweight was 42.3%, and obesity 20.9%. For men, the prevalence was 47.6% and 19.6%, and for women 37.8% and 22.1%, for overweight and obesity, respectively. Only 7.2% of the total sample presented five healthy lifestyle features—among men the prevalence was 6.8%, and among women it was 7.6% (Table 1).

The prevalence of healthy behaviors according to BMI categories is presented in Table 2. Most participants with normal weight were physically active, ate more fruit and vegetables, did not drink nor smoke, and presented a healthy lifestyle, than those who were overweight or obese. Similarly, a greater percentage of participants with normal weight and overweight reported having a rested sleep than their obese peers.

The relationship between health behaviors with overweight and obesity are presented in Table 3. In general, the models presented appropriate goodness-of-fit values, except for the model of the association between alcohol consumption and obesity. Having a healthy lifestyle (five healthy lifestyle behaviors) was found to halve the odds of being obese (OR = 0.50, 95% CI: 0.39–0.63), however, it was not associated with overweight (OR = 0.93, 95% CI: 0.79–1.10). The health behavior that presented the strongest correlation was physical activity participation, as older adults who complied with the recommendations (≥5 days/week) had 42% lower odds of being obese (OR = 0.58, 95% CI: 0.52–0.66). Those who reported not having a restless sleep (OR = 0.62, 95% CI: 0.55–0.70) and eating fruit and vegetables (≥4 portions/day) (OR = 0.82, 95% CI: 0.72–0.93) had fewer odds of being obese. Alcohol consumption was associated with neither overweight nor obesity. Not smoking was inversely associated with both overweight and obesity (OR = 1.35, 95% CI: 1.18–1.55 and OR = 1.36, 95% CI: 1.14–1.61, respectively).

Sex moderated the association of fruit and vegetable consumption, alcohol use, and smoking with obesity—multivariate logistic regression (Table 4), adjusted for age, civil status, education level, and living place, analyzed by sex, found that having the five healthy lifestyle behaviors was significantly associated with fewer odds of obesity (men: OR = 0.59, 95% CI: 0.41–0.84 and women: OR = 0.45, CI: 0.33–0.63), but not overweight (men: OR = 0.97, 95% CI: 0.76–1.24 and women: OR = 0.91, 95% CI: 0.73–1.13). For men, being physically active (OR = 0.59, 95% CI: 0.50–0.71), having a good sleep (OR = 0.60, 95% CI: 0.49 to 0.74), and not drinking excessively alcohol (OR = 0.59, 95% CI: 0.44–0.79) was associated with lower odds of being obese. For women, being physically active (OR = 0.58, 95% CI: 0.49–0.68), complying with the fruit and vegetable recommendations (OR = 0.75, 95% CI: 0.63–0.88), having good sleep (OR = 0.63, 95% CI: 0.54–0.73) was significantly associated with lower odds of obesity. Not smoking was significantly associated with increased odds of overweight and obesity for women (OR = 1.43, 95% CI: 1.15–1.77 and OR = 1.70, 95% CI: 1.30–2.23, respectively). The models stratifying by sex also presented appropriate goodness-of-fit, with the exception of the association between alcohol consumption and overweight as well as the association between smoking and obesity among women.

## 4. Discussion

This study aimed to analyse the association of healthy lifestyle behaviors with weight status among European older adults. Having five healthy lifestyle behaviors decreased by 50% the odds of being obese. The more noticeable health behavior was physical activity, in which attending the recommendations reduced by 42% the odds of obesity, followed by sleep quality and eating fruit and vegetables according to recommendations. These results suggest that obesity in older adults is a complex condition with physical activity making a key contribution. In this study, for the total sample alcohol consumption did not present a significant association with obesity and smoke had an inverse association, i.e., not smoking increased the odds of being obese by 36%. Also, we found that sex moderated the association of fruit and vegetable consumption, alcohol use, and smoking with obesity. Women who attended the recommend of physical activity, consumed recommended portions of fruit and vegetable and had a rested sleep presented lower odds of obesity, while women that did not smoke presented higher odds of obesity. Men who attended the recommendation of physical activity, had a rested sleep and did not drink alcohol excessively presented lower odds of obesity.

It is important to highlight that we found a significant association of healthy lifestyle behaviors with obesity but not with overweight, except for smoking, which presented an inverse association, not smoking increased the odds of being overweight by 35% and to be obese by 36%. A cross-sectional study, with a sample from 31 countries, found a significant association of healthy lifestyle behavior with obesity and overweight but in younger samples (mean age 46.6 ± 17.4 years) [10]. Among men and women, ex-smoking was positively associated with both overweight and obesity, and moderate alcohol use was positively associated with both overweight and obesity. Daily consumption of fruit and vegetables was found to be protective from both overweight and obesity, overall and for men but not for women. Physical activity was positively associated with overweight but not obesity [10].

Comparing our analyses with the study of the prevalence of healthy lifestyle behavior in Europeans of all ages (≥18 years, only 5.8% presented five healthy lifestyle behaviors) [13], it seems that older adults are more prone to fill the recommendations, even if the value remains extremely low (7.3%). With North Americans, 7.7% reported pursuing a healthy lifestyle behavior, although the type of measure was slightly different [21]. They considered four health behaviors—nonsmoking, healthy weight, eating five fruit and vegetable portions per day and doing physical activity (≥30 min for ≥5 times per week).

The recent progress pertaining to therapeutic approaches to obesity in older adults is lifestyle intervention incorporating caloric restriction and exercise consisting of aerobic endurance training and resistance training [22]. Therefore, our study supports the need for lifestyle interventions, considering that our main finding was that engagement in ≥5 days/week physical activity is the most important behavior to prevent obesity. Physical activity is considered an important component of weight control and it is the main recommendation in European Guidelines to manage obesity, besides diet [23]. In addition to weight control, physical activity reduces the prevalence of vulnerability, e.g., in terms of chronic conditions [24] and mortality rates [25] in older adults. The recent European recommendation for old adults’ exercise is to do varied multicomponent physical activity that emphasizes functional balance and strength training at moderate or greater intensity on three or more days a week, to enhance functional capacity and to prevent falls [26]. Despite the benefits of physical activity, habitual physical activity declines with aging [27]. Thus, it is essential to encourage health programs that assist the practice of physical exercise for this population. In addition, exercise (beyond its effects regarding an improvement in weight loss) also improves the quality of sleep [28].

Our study shows that the second healthy lifestyle behavior that was most associated with obesity was sleep quality. Sleep without restlessness decreased the odds of obesity by 38%. This is in line with other studies, which found that reduced sleep durations are strongly associated with greater adiposity in older adults [29]. Sleep duration of less than 5 h was associated with a body mass index (BMI) that was on average 2.5 kg/m^2^ greater in men and 1.8 kg/m^2^ greater in women [29]. Studies have been reinforcing the importance of sleep quality to health and even associated it with mortality [30,31]. Both short and long duration of sleep are significant predictors of death in prospective population studies [30]. Also, short sleep duration has been linked to hypertension, diabetes, and cardiovascular disease [32]. All those are risk factors of obesity.

Malnutrition is one of the highest risks to the health, autonomy, and well-being of older adults. A decline in energy and protein intake is frequently observed with aging, in particular for the very old and/or dependent people [33]. Fruits and vegetables are well-appreciated but the consumption remains insufficient for older adults [34]. A multicenter study with Europeans old adults (65–79 years) showed that nutrition-related knowledge and nutrition-related attitudes were associated with lower BMI and higher physical activity [35]. In our study, only 21% in the obese category consumed four or more portions of fruit and/or vegetable per day. For women who comply with the recommendation, it was associated with fewer odds of being obese. Because controlled energy intake is essential to weight management, it is important to encourage older adults to increase fruit and vegetable consumption, which have high water content and are sources of vitamins, minerals, and fiber and can increase satiety for a longer time [36].

Being a former smoker was positively associated with overweight and obesity in prior research [10]. Also, considering older adults, our findings somewhat agree with previous findings, in which older adults that were current smokers presented lower odds of obesity. However, other factors can change this association, as heavy smokers also presented a higher risk of obesity [37]. Nicotine withdrawal seems to be associated with hunger and food consumption, and smokers who quit gain approximately 10 pounds within the first years of abstinence [38]. Our study did not consider if an individual stopped smoking or never smoked, as we recorded smoking habits into two categories, current smoker (regularly or sometimes) and not smoking (never smoked or stop smoking). Possibly, for this reason, not smoking was significantly associated with overweight and obesity. Nevertheless, more investigation is needed regarding obesity and smoke behavior, particularly for the older population.

Alcohol consumption and weight status presented variations by sex, with a positive association with obesity for men and a null association for women. The same was found in other studies [10]. However, our study considered that light drinking (1 to 7 units/week) and moderate drinking (7 to 14 units/week for women and 7 to 21 units/week for men) are in the same range. Other studies found that light-to-moderate alcohol intake was protective to gain weight and higher levels of drinking were associated with weight gain, despite the sample being younger [39,40].

Despite the association of each health behavior with overweight and obesity, which have distinct mechanisms and characteristics, our findings highlighted that the clustering of healthy behaviors did not provide additional protection for overweight and obesity than isolated healthy behaviors. This finding can be explained possibly due to the strong association between healthy behaviors among older adults [41,42], in which different health behaviors can have a synergic association with overweight and obesity, highlighting once again the importance of interventions integrating different health behaviors in older adults.

This study has some limitations that should be considered. One is that weight and height were self-reported and older adults have certain limitations to refer the exact values, overestimating the height and subestimating the weight. Second, BMI is not the best measure to analyze overweight and obesity, especially in the older population. In general, measurements of body weight and height are used to reflect body fat in large (epidemiological) studies or in clinic settings [2,11], as such measurements provide a rapid and cheap way to estimate body fatness [43]. However, aging induces changes in body composition, such as a redistribution of fat from peripheral and subcutaneous sources to a central location, leading to increased waist circumference and waist–hip ratio in older adults [44]. Therefore, BMI cannot fully reflect muscle mass and body fat [45] and should be analyzed with some caution in the older population. Another limitation is the fact that we divided the healthy lifestyle behaviors into two categories, and could not identify more subtle changes, especially regarding smoke habits and alcohol consumption. Furthermore, this study has a cross-sectional design and does not allow causal conclusions.

## 5. Conclusions

Obesity is a complex pathology to be analyzed in the older population. Understanding lifestyle behaviors that are associated with overweight and obesity in the older population could contribute to implement more effective health programs to prevent or treat obesity and related vulnerability in old age. The present study concludes that physical activity may have a key role in preventing obesity and should be carried out by the recommendations for practice. In addition to that, the quality of sleep and the intake of fruits and vegetables may contribute to reduce the risk of obesity. Our study is in accordance with the most recent recommendation for obesity in older adults [22], which is an integrated approach; clinical practice should incorporate lifestyle interventions. It is important to incorporate possible sex differences regarding specific behaviors, such as with respect to fruit and vegetable and alcohol consumption. The association of alcohol consumption and smoking habits with obesity needs more research, especially for older populations. Future studies could also present a longitudinal design, so causal conclusions could be drawn.

## Figures and Tables

**Table 1:** Participants’ characteristics for the total sample.

	Total (*n* = 8938) % or M (95% CI)	Men (*n* = 4099) % or M (95% CI)	Women (*n* = 4839) % or M (95% CI)
Age	73.6 (73.5 to 73.7)	73.2 (73.0 to 73.4)	73.9 (73.7 to 74.1)
Education			
Low	25.3 (24.4 to 26.2)	19.7 (18.5 to 20.9)	30.0 (28.7 to 31.3)
Middle	51.7 (50.7 to 52.8)	57.8 (56.3 to 59.3)	46.6 (45.2 to 48.0)
High	23.0 (22.2 to 23.9)	22.5 (21.3 to 23.8)	23.4 (22.3 to 24.6)
Marital status			
Live with partner	56.6 (55.5 to 57.6)	71.9 (70.5 to 73.3)	43.6 (42.2 to 45.0)
Live without a partner	43.4 (42.4 to 44.5)	28.1 (26.7 to 29.5)	56.4 (55.0 to 57.8)
Living place			
Urban	63.8 (62.8 to 64.8)	62.0 (60.5 to 63.4)	65.4 (64.0 to 66.7)
Rural	36.2 (35.0 to 37.2)	38.0 (36.3 to 39.3)	34.6 (33.1 to 35.8)
BMI (kg/m^2^)			
Normal (18.5–24.9)	36.8 (35.8 to 37.8)	32.8 (31.4 to 34.3)	40.1 (38.8 to 41.5)
Overweight (25–29.9)	42.3 (41.3 to 43.3)	47.6 (46.1 to 49.2)	37.8 (36.4 to 39.2)
Obese (≥30)	20.9 (20.1 to 21.8)	19.6 (18.4 to 20.8)	22.1 (20.9 to 23.3)
Physical activity ≥ 5 days/week			
No	67.8 (66.8 to 68.7)	65.5 (64.0 to 66.9)	69.7 (68.4 to 71.0)
Yes	32.2 (31.3 to 33.2)	34.5 (33.1 to 36.0)	30.3 (29.0 to 31.6)
Fruit/vegetable ≥ 4 portions/day			
No	76.7 (75.8 to 77.5)	80.5 (79.3 to 81.7)	73.4 (72.1 to 74.6)
Yes	23.3 (22.5 to 24.2)	19.5 (18.3 to 20.7)	26.6 (25.4 to 27.9)
Sleep was rested			
No	20.3 (19.5 to 21.1)	15.2 (14.1 to 16.3)	24.7 (23.5 to 25.9)
Yes	79.7 (78.9 to 80.5)	84.8 (83.7 to 86.0)	75.3 (74.1 to 76.5)
Drink excessively			
Yes	4.2 (3.8 to 4.7)	5.6 (4.9 to 6.4)	3.1 (2.6 to 3.6)
No	95.8 (95.3 to 96.2)	94.4 (93.6 to 95.1)	96.9 (96.4 to 97.4)
Smoking			
Yes	11.3 (10.7 to 12.0)	14.2 (13.2 to 15.3)	8.9 (8.1 to 9.7)
No	88.7 (88.0 to 89.3)	85.8 (84.7 to 86.8)	91.1 (90.3 to 91.9)
Healthy lifestyle behaviors			
None	0.2 (0.1 to 0.3)	0.2 (0.1 to 0.5)	0.1 (0.0 to 0.3)
1 behavior	2.7 (2.4 to 3.1)	3.4 (2.9 to 4.0)	2.2 (1.8 to 2.6)
2 behaviors	17.0 (16.2 to 17.8)	16.2 (15.1 to 17.4)	17.6 (16.6 to 18.7)
3 behaviors	44.6 (43.6 to 45.6)	44.2 (42.7 to 45.7)	44.9 (43.5 to 46.3)
4 behaviors	28.3 (27.3 to 29.2)	29.1 (27.7 to 30.5)	27.5 (26.3 to 28.8)
5 behaviors	7.2 (6.7 to 7.8)	6.8 (6.1 to 7.6)	7.6 (6.9 to 8.4)

Abbreviation: BMI, body mass index. M: median; CI: Confidence Interval.

**Table 2:** Prevalence of healthy lifestyle behavior among all participants by body mass index.

	Normal Weight % (95% CI)	Overweight % (95% CI)	Obese % (95% CI)
PA ≥ 5 days/week			
No	63.4 (61.7 to 65.0)	67.6 (66.1 to 69.0)	75.9 (73.9 to 77.8)
Yes	36.6 (35.0 to 38.3)	32.4 (31.0 to 33.9)	24.1 (22.2 to 26.1)
Fruit/vegetable ≥ 4 portions/day			
No	73.8 (72.2 to 75.3)	78.0 (76.6 to 79.3)	79.0 (77.1 to 80.8)
Yes	26.2 (24.7 to 27.8)	22.0 (20.7 to 23.4)	21.0 (19.2 to 22.9)
Sleep was rested			
No	17.8 (16.5 to 19.1)	19.2 (18.0 to 20.5)	26.9 (24.9 to 28.9)
Yes	82.2 (80.9 to 83.5)	80.8 (79.5 to 82.0)	73.1 (71.1 to 75.1)
Drink excessively			
Yes	3.7 (3.2 to 4.3)	4.3 (3.7 to 5.0)	5.1 (4.2 to 6.2)
No	96.3 (95.7 to 96.9)	95.7 (95.0 to 96.3)	94.9 (93.8 to 95.8)
Smoking			
Yes	13.6 (12.5 to 14.8)	10.1 (9.1 to 11.1)	9.8 (8.5 to 11.2)
No	86.4 (85.2 to 87.5)	89.9 (88.9 to 90.9)	90.2 (88.8 to 91.5)
Healthy lifestyle behavior			
None	0.2 (0.0 to 0.4)	0.1 (0.1 to 0.3)	0.3 (0.1 to 0.7)
1 behavior	2.9 (2.4 to 3.5)	2.0 (1.6 to 2.5)	4.0 (3.2 to 4.9)
2 behaviors	15.0 (13.8 to 16.3)	16.6 (15.5 to 17.9)	21.1 (19.3 to 23.0)
3 behaviors	42.2 (40.5 to 43.8)	46.2 (44.7 to 47.8)	45.6 (43.3 to 47.9)
4 behaviors	30.5 (29.0 to 32.2)	28.0 (26.6 to 29.4)	24.7 (22.8 to 26.8)
5 behaviors	9.2 (8.3 to 10.3)	7.0 (6.2 to 7.8)	4.3 (3.4 to 5.3)

Abbreviation—PA, physical activity. CI: Confidence Interval.

**Table 3:** Adjusted odds ratios for the relationship between health behaviors with overweight and obesity among adults >65 years.

Characteristics	Overweight	Sex-Interaction	Obesity	Sex-Interaction
	OR (95% CI)	OR (95% CI)	OR (95% CI)	OR (95% CI)
Physical activity ≥ 5 days/week				
No	Ref	Ref + male	Ref	Ref + male
Yes	0.98 (0.90–1.08)	0.98 (0.82–1.18)	0.58 (0.52–0.66)	0.93 (0.74–1.19)
Fruit/vegetable ≥ 4 portions/day				
No	Ref	Ref + male	Ref	Ref + male
Yes	0.90 (0.82–1.00)	0.92 (0.75–1.13)	0.82 (0.72–0.93)	0.76 (0.59–0.98)
Sleep was rested				
No	Ref	Ref + male	Ref	Ref + male
Yes	1.05 (0.94–1.16)	0.85 (0.68–1.060	0.62 (0.55–0.70)	1.00 (0.78–1.28)
Drink excessively				
Yes	Ref	Ref + male	Ref	Ref + male
No	1.05 (0.85–1.30)	0.86 (0.56–1.33)	0.81 (0.64–1.03)	2.45 (1.46–4.13)
Smoking				
Yes	Ref	Ref + male	Ref	Ref + male
No	1.35 (1.18–1.55)	1.10 (0.83–1.45)	1.36 (1.14–1.61)	1.55 (1.09–2.19)
5 healthy lifestyle behaviors				
No	Ref	Ref + male	Ref	Ref + male
Yes	0.93 (0.79–1.10)	0.93 (0.67–1.29)	0.50 (0.39–0.63)	0.75 (0.46–1.21)

Note. Adjusted for sex, age, civil status, education level, and living place. Sex-interactions adopted men as reference. Reference for the interactions was the same reference of the models without interactions and men. OR, odds ratio. CI, confidence interval. Hosmer–Lemeshow test—Overweight: physical activity: 11.0, *p* = 0.204. Fruit/vegetable consumption: 3.1, *p* = 0.931. Sleep: 8.5, *p* = 0.389. Alcohol consumption: 11.1, *p* = 0.195. Smoking: 2.0, *p* = 0.981. 5 healthy lifestyle behaviors: 8.1, *p* = 0.428. Obesity—physical activity: 9.3, *p* = 0.315. Fruit/vegetable consumption: 12.0, *p* = 0.150. Sleep: 4.5, *p* = 0.811. Alcohol consumption: 16.0, *p* = 0.042. Smoking: 11.9, *p* = 0.155. 5 healthy lifestyle behaviors: 15.4, *p* = 0.052.

**Table 4:** Adjusted odds ratios for overweight and obesity among older adults for men and women.

Characteristics	Overweight		Obesity	
	Men OR (95% CI)	Women OR (95% CI)	Men OR (95% CI)	Women OR (95% CI)
PA ≥ 5 days/week				
No	Ref	Ref	Ref	Ref
Yes	0.99 (0.87–1.13)	0.98 (0.86–1.11)	0.59 (0.50–0.71)	0.58 (0.49–0.68)
Fruit/vegetable ≥ 4 portions/day				
No	Ref	Ref	Ref	Ref
Yes	0.95 (0.81–1.10)	0.88 (0.77–1.00)	0.96 (0.79–1.17)	0.75 (0.63–0.88)
Sleep was rested				
No	Ref	Ref	Ref	Ref
Yes	1.16 (0.97–1.37)	0.99 (0.86–1.13)	0.60 (0.49–0.74)	0.63 (0.54–0.73)
Drink excessively				
Yes	Ref	Ref	Ref	Ref
No	1.12 (0.87–1.46)	0.96 (0.69–1.34)	0.59 (0.44–0.79)	1.40 (0.91–2.16)
Smoking				
Yes	Ref	Ref	Ref	Ref
No	1.29 (1.08–1.55)	1.43 (1.15–1.77)	1.14 (0.91–1.43)	1.70 (1.30–2.23)
5 healthy lifestyle behaviors				
No	Ref	Ref	Ref	Ref
Yes	0.97 (0.76–1.24)	0.91 (0.73–1.13)	0.59 (0.41–0.84)	0.45 (0.33–0.63)

Note. PA, physical activity. OR, odds ratio. CI, confidence interval. Adjusted for age, civil status, education level, and living place. Hosmer–Lemeshow test: Overweight—Men: physical activity: 2.9, *p* = 0.940. Fruit/vegetable consumption: 3.6, *p* = 0.892. Sleep: 12.7, *p* = 0.124. Alcohol consumption: 2.7, *p* = 0.954. Smoking: 5.0, *p* = 0.761. 5 healthy lifestyle behaviors: 4.8, *p* = 0.780. Women: physical activity: 10.0, *p* = 0.263. Fruit/vegetable consumption: 11.5, *p* = 0.177. Sleep: 12.8, *p* = 0.120. Alcohol consumption: 18.0, *p* = 0.022. Smoking: 8.6, *p* = 0.375. 5 healthy lifestyle behaviors: 11.9, *p* = 0.156. Obesity—Men: physical activity: 5.2, *p* = 0.741. Fruit/vegetable consumption: 10.3, *p* = 0.244. Sleep: 6.7, *p* = 0.566. Alcohol consumption: 6.9, *p* = 0.551. Smoking: 6.4, *p* = 0.602. 5 healthy lifestyle behaviors: 8.2, *p* = 0.412. Women: physical activity: 6.3, *p* = 0.615. Fruit/vegetable consumption: 11.7, *p* = 0.168. Sleep: 10.6, *p* = 0.227. Alcohol consumption: 10.8, *p* = 0.215. Smoking: 19.2, *p* = 0.014. 5 healthy lifestyle behaviors: 13.0, *p* = 0.111.

## Data Availability

The European Social Survey data are open access and accessible at: https://www.europeansocialsurvey.org/data/.
